# Surfactant mediated liquid phase exfoliation of graphene

**DOI:** 10.1186/s40580-015-0050-x

**Published:** 2015-10-08

**Authors:** Rekha Narayan, Sang Ouk Kim

**Affiliations:** Department of Materials Science & Engineering, KAIST, Daejeon, 305-701 Republic of Korea

**Keywords:** Graphene, Exfoliation, Surfactant, Dispersion, Solvent

## Abstract

Commercialization of graphene based applications inevitably requires cost effective mass production. From the early days of research on graphene, direct liquid phase exfoliation (LPE) of graphite has been considered as the most promising strategy to produce high-quality mono or few-layer graphene sheets in solvent dispersion forms. Substantial success has been achieved thus far in the LPE of graphene employing numerous solvent systems and suitable surfactants. This invited review article principally showcase the recent research progress as well as shortcomings of surfactant assisted LPE of graphene. In particular, a comprehensive assessment of the quality and yield of the graphene sheets produced by different categories of the surfactants are summarized. Future direction of LPE methods is also proposed for the eventual success of commercial applications.

## Introduction : liquid phase exfoliation of graphene – advantages and challenges

Graphene has been the most sensational material discovery over the past decades along with its unprecedented material properties such as ultrahigh tensile strength (~1TPa), high thermal conductivity of (5,000 W m^−1^ K^−1^), large specific surface area (2,630 m^2^ g^−1^), ballistic electron mobility (250,000 cm^2^V^−1^ s^−1^) and optical transparency (97.7 %) [1–6]. As a result of the worldwide boom in graphene research, a wide range of applications have been explored, including flexible/stretchable devices [7–9], high-frequency transistors [10, 11], energy storage/conversion [12], sensors [13], biomedical applications [14], and composites [15]. Despite numerous research efforts, nonetheless, the discovery still seems far from commercial reality, which is principally due to the limited scalability and high cost of currently available graphene production methods.

Graphene production methods can be classified into top-down and bottom-up approaches. Well-known top-down methods include (i) mechanical exfoliation (Scotch tape method) historically used in the first discovery of graphene by Geim and Novoselov [1], (ii) chemically converted graphene (reduction of graphene oxide) [16], (iii) electrochemical exfoliation [17], (iv) liquid phase exfoliation (LPE) in the presence/absence of surfactants [18] and so on. Bottom-up approaches synthesize mono or few layer graphene structures from small molecule organic precursors by catalytic chemical vapour deposition (CVD) or organic synthesis or epitaxial growth on SiC and so on. Presently, reduction of chemically exfoliated graphene oxide is the most popular strategy for bulk graphene production among the aforementioned various approaches. Unfortunately, post-reduction methods cannot completely cure the structural defects introduced by the strong oxidation process. Thus, the band structure and electronic properties unique to graphene are severely deteriorated.

From early days of graphene research, LPE has been anticipated as the most desirable mass-production method for graphene. The principal attraction of this method is that, it is a straightforward and scalable process where pristine graphite or expandable graphite (obtained by thermal or microwave expansion of graphite intercalation compounds) is directly subjected to a solvent treatment to weaken the van der Waals attractive forces between graphene interlayers. External driving force such as ultrasonication, electric field or shearing can be applied to facilitate the spontaneous exfoliation into graphene sheets. Another significant advantage of this method is the production of exfoliated graphene sheets in the form of solvent suspension that allows an immediate utilization for spin-coating, spray painting or any other solution processing. For instance, simple vacuum filtration of the as-obtained graphene suspensions can be used for the fabrication of thin films with high conductivities [19]. Novel graphene/polymer composites can be easily prepared by direct solution mixing. As such, LPE method addresses all crucial prospects for viable industrial applications.

Graphene is known to suffer from only a limited solvent dispersibility even for its good solvents, such as DMF or NMP, which is due to the small mixing entropy gain and strong intersheet π-π attraction of the generic two-dimensional structure. Moreover, those good solvents are toxic, expensive and not so volatile such that solution processing from those solvents is practically challenging. Alternative route is to employ an appropriate surfactant, which can mediate dispersion in water or any other mild volatile solvents. To date, a variety of surfactants belonging to different categories, including ionic /non-ionic, aromatic/non-aromatic, polymeric etc. have been investigated. However, these researches require further optimization for practical use and it is highly recommended to understand surfactant-solvent interaction in a more systematic way. To this end, this review article is motivated to offer an overview on the state-of art of LPE of graphene with the prime focus on surfactant-assisted exfoliation. In the first part of this article, we will briefly discuss the key parameters involved in the optimization of a fruitful LPE recipe. The subsequent sections will provide a systematically categorized comprehensive discussion on the recent progress in the surfactant promoted LPE of graphene.

## Review : liquid phase exfoliation – key factors

### Dispersing medium : solvent

In an LPE recipe, solvent is the most important factor dominating the overall productivity of exfoliation. An ideal solvent should be able to effectively overcome the van der Waals interaction between the graphene layers held within a π-π stacking distance of 3.35-3.4 Å. In 2008, two independent groups of Coleman et al. and Novoselov et al. reported a significant discovery that graphite crystals could be directly exfoliated in certain organic solvents to give defect free monolayer graphene [19, 20]. In the presence of a solvent, the potential energy between the adjacent layers given by the dispersive London forces becomes significantly reduced. Coleman et al. proposed that when the refractive index of solvent matches with that of graphene, this potential energy can even approach zero. They demonstrated that solvents with interfacial tension (γ) around ~41 mJm^−2^ is desired to minimize the energy input in attaining effective separation of sheets beyond the range of the strong van der Waals forces [19]. An approximate expression from a thermodynamic perspective was also provided to account for their experimental results as given below.

$$ \frac{H_{mix}}{V_{mix}}=\frac{2}{T_{NS}}{\left(\sqrt{E_{SS}}-\sqrt{E_{SG}}\right)}^2{\varPhi}_G $$

where ∆H_mix_ is the enthalpy of mixing, V_mix_ is the volume of the mixture, T_NS_ the thickness of graphene nanosheet, E_SS_ and E_SG_ are the surface energies of solvent and graphene, respectively, ɸ_G_ is the volume fraction of graphene dispersed. Accordingly, solvents belonging to this category, including N-methylpyrrolidone (NMP) (γ = 40 mJ m^−2^), N,N’-dimethylformamide (DMF) (γ = 37.1 mJ m^−2^) and ortho-dichlorobenzene (o-DCB) (γ = 37 mJ m^−2^) have been widely employed for LPE of graphene.

**Fig. 1:**
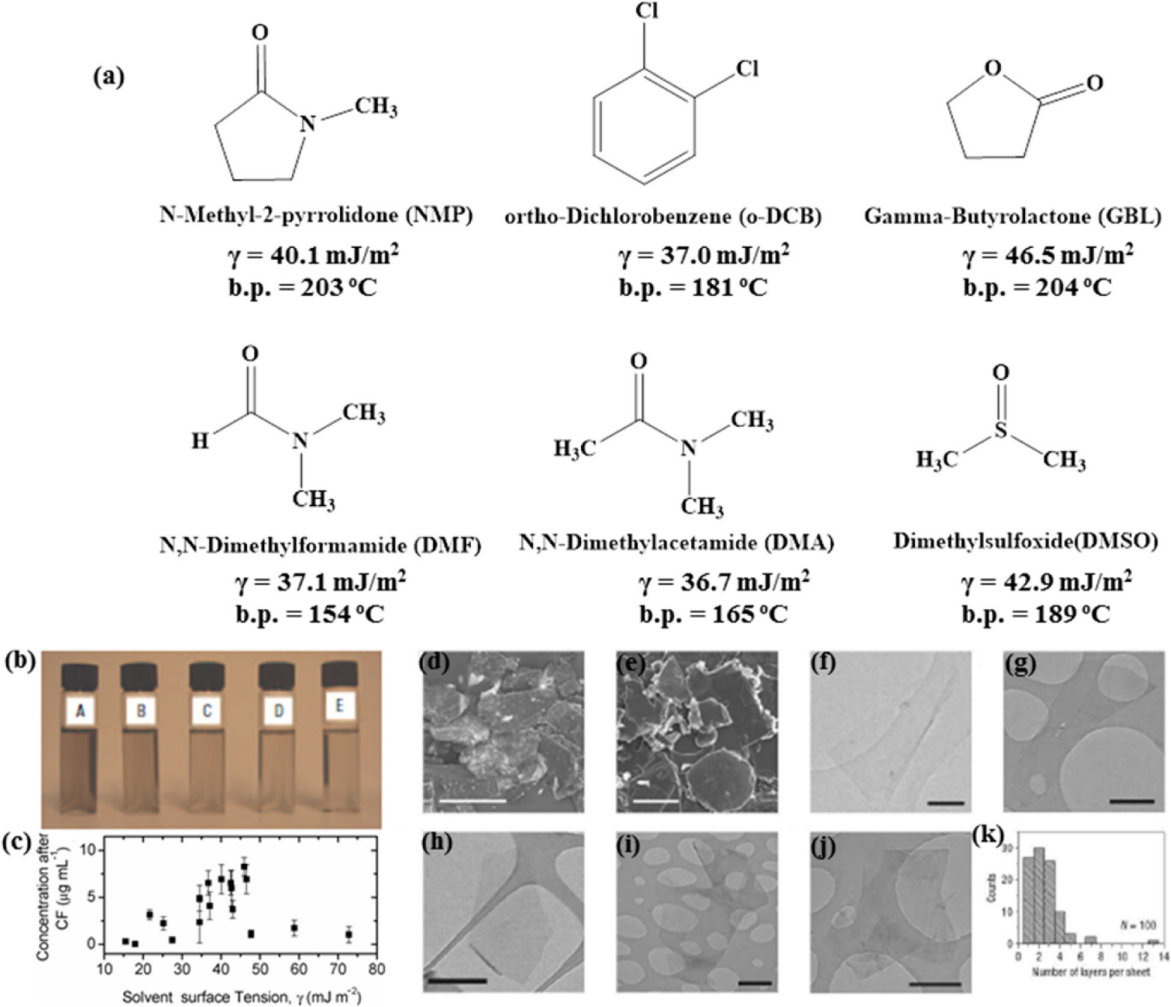
Solvents for LPE of graphene. **a** Chemical structures of common organic solvents used in LPE, along with their surface tension and boiling points. **b** Graphene dispersion in NMP after centrifugation at 6–4 μg/mL concentrations (**a**) to (**e**). **c** Dispersed graphene concentration as a function of solvent surface tension/energy. **d** SEM image of pristine graphite (scale bar: 500 μm). **e** SEM image of sediment after centrifugation (scale bar: 25 μm). (**f**-**h**) Bright field TEM images of monolayer graphene sheets deposited from GBL (**f**), DMEU (**g**) and NMP (**h**) (Scale bar : 500 nm). (i, j) Bright field TEM images of a folded and multilayer graphene sheets respectively, deposited from NMP (scale bar: 500 nm). **k** Histogram of the number of graphene layers per flakes for NMP dispersions. **b**-**k** reproduced from ref. 19 with permission, © Nature Publishing Group)

Figure 1(a) displays a few commonly utilized solvents for graphene exfoliation along with their surface tensions as well as boiling points. Among the large variety of solvents explored, the most successful results have been achieved with an organic solvent, NMP [19]. This solvent was reported to produce minimal oxidized exfoliated sheets with approximately 28 % monolayer flakes and above 75 % sheets with less than 6 layer thickness. Unfortunately, the yield was low at ~1 wt % and the maximum lateral dimension of graphene was on the order of a few microns (Fig. 1(b)-(e)). A serial re-sonication/re-centrifugation strategy of the unexfoliated sediment was recommended to increase the yield. The thin film prepared from these sheets exhibited the electrical conductivity of ~6500 Sm^−1^.

Ortho-dichlorobenzene (o-DCB) was shown to be another fair solvent for graphite exfoliation giving a dispersibility range of 0.03 mg/mL [21]. Following the trend, Bourlinos et al. in 2009 explored a series of electron deficient perfluorinated aromatic solvents such as hexafluorobenzene(C_6_F_6_), octafluorotoluene (C_6_F_5_CF_3_), pentafluorobenzonitrile (C_6_F_5_CN), and pentafluoropyridine (C_5_F_5_N) to exfoliate fine graphite powder within relatively short sonication period of 1 h. Maximum dispersion concentration upto 0.1 mg/mL were obtained with pentafluorobenzonitrile, whereas the poorest yield of 0.05 mg/mL was measured for octafluorotoluene as well as pentafluoropyridine [22], Inspired by Coleman’s approach, Tagmatarchis and co-workers accomplished efficient exfoliation of graphite flakes in benzylamine solvent for prolonged sonication periods of 4–6 h leading to improved few-layer graphene dispersion concentration ~ 0.5 mg/mL [23]. Further increase of the sonication period beyond 10 h did not seem to cause any increase in dispersion concentration. In a very recent study, to overcome the low yield and poor exfoliation issues, Sun et al. introduced four amine based organic solvents, namely 3,30-iminobis (N,Ndimethylpropylamine) (DMPA), N-[3-(dimethylamino)propyl]methacrylamide (DMAPMA), 2-(tert-butylamino) ethyl methacrylate (BAEMA) and 2-(dimethylamino) ethyl methacrylate (MAEMA), which challenge to outperform the previously known the best solvent, NMP, and other LPE systems with surfactants, including sodium cholate (SC), sodium taurodeoxycholate (STC) and polyvinylpyrrolidone(PVP) [24]. In their control experiments, in particular DMPA exhibited 1.5 times higher dispersing capacity than NMP. Further optimization of the process was done using pre-exfoliated graphene as a starting material, obtained from 12 h bath sonication in isopropanol. This promoted the final graphene concentration up to ~1.4 mg/mL with a yield of 14 %. Spontaneous exfoliation of HOPG in chlorosulfonic acid was achieved by Behabtu et al. to produce a high concentration dispersion of monolayer sheets upto 2 mg/mL [25].

Majority of the above discussed solvents, even though successful to large extent, have significant drawbacks that limit the scalability for industrial manufacture. Solvents like NMP, DMF etc. are very expensive as well as highly toxic. In particular, NMP is regarded as a potential human reproductive hazard, which is easily absorbed through skin. Moreover, these solvents have high boiling points (NMP, 203 °C), making it difficult to deposit the exfoliated graphene flakes onto a target substrate. This would be a critical drawback in the fabrication of graphene transparent conductor for solar cells [26], field effect transistors [27], photodetectors [28] and so on. As those solvents take significant time for evaporation, re-aggregation of exfoliated graphene sheets may easily occur. Therefore, it is of paramount significance to explore more volatile and less toxic solvents along with the superior dispersing capability. To this end, attempts had been made to transfer graphene dispersions in NMP to low boiling solvents like ethanol via solvent exchange, noteworthy the sample showed 20 % sedimentation within one week [29]. Nonetheless, direct graphene exfoliation in a single low boiling solvent is always preferable owing to the simplicity of process. Catheline et al. applied volatile THF (tetrahydrofuran) to produce graphenide solutions (solutions of negatively charged graphene flakes) by dissolution of graphite intercalation compound (GIC) KC8 [30]. Severely crumpled graphene sheets were obtained that made it difficult to determine the precise thickness. A solvothermal-assisted exfoliation of expanded graphite in acetonitrile was attempted by Hou et al. utilizing the dipole-induced dipole interactions between graphene and polar acetonitrile [31]. Coleman et al. investigated some common volatile solvents like chloroform, isopropanol and acetone as exfoliating media for graphene, but longer sonication times around 360 h were required to produce dispersions with the concentrations of 0.4 and 0.5 mg/mL from chloroform and isopropanol respectively [32].

### External forces : ultrasonication/shear mixing

**Fig. 2:**
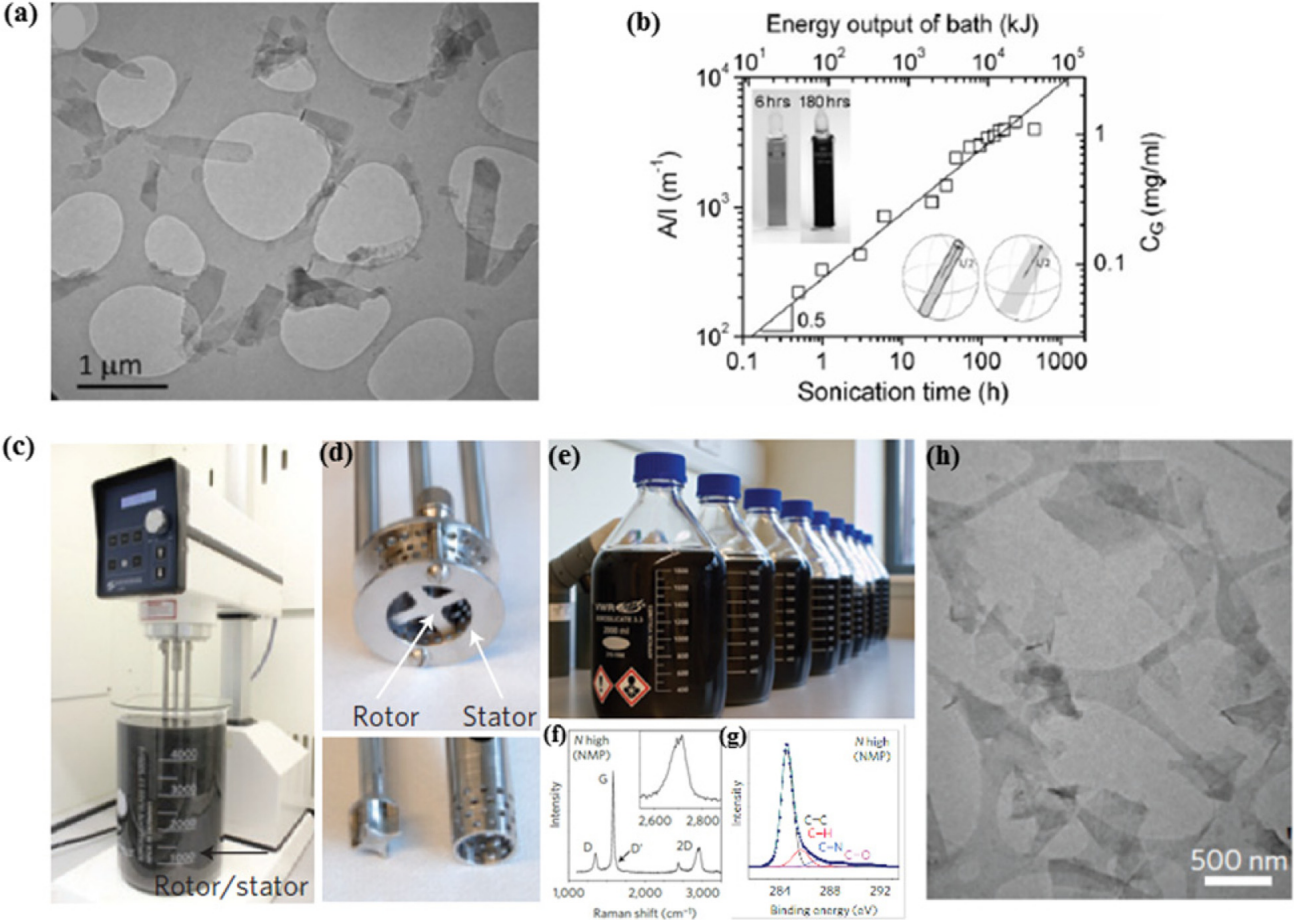
Sonication/Shear forces in LPE. **a** Broad-field TEM image showing the small flakes observed after long sonication times (180 h). **b** Concentration of graphene after centrifugation as a function of sonication time. The left axis shows the measured absorbance per cell length, A/l, while the right axis shows the concentration calculated using an absorption coefficient of 3620 mL mg^−1^ m^−1^. The line illustrates √t behavior. The upper axis shows the total energy output of the bath calculated using the measured power output of 23 W. **a**-**b** reproduced from ref. 34 with permission, © Wiley-VCH). **c** A Silverson model L5M high-shear mixer with mixing head in a 5 *l* beaker of graphene dispersion. **d** Close-up view of a *D*D32mm mixing head and a *D*D16mm mixing head with rotor (left) separated from stator. **e** Graphene-NMP dispersions produced by shear exfoliation. (f,g) The presence of monolayers confirmed by Raman (**f**) and XPS (**g**) spectra (NMP-shear exfoliated samples). **h** Wide-field TEM image of SEG nanosheets (after centrifugation). **c**-**h** reproduced from ref. 36 with permission, © Nature Publishing Group)

LPE of graphite is commonly accompanied by external forces such as ultrasonication or shear mixing. While ultrasonication sound waves produce strong compression and rarefaction, the resultant vacuum cavities in the medium collapse and generate high pressure jets that can peel off the graphene layers from graphite. The attractive van der Waals forces between the adjacent graphene layers can be significantly weakened by increasing the π-π stacking distance (r) beyond 5 Å, as the van der Waals force is proportional to 1/r^6^ [33]. Ultrasonication or shear force may greatly help the intercalation of solvent molecules into bulky graphite layers; thereby effectively increase the interlayer spacing for the eventual exfoliation of mono- and/or multi-layered graphene sheets. In order to improve the yields of exfoliation, a widely used strategy is to drastically increase the sonication times. For instance, low power bath sonication of graphite powder in NMP for 460 h yielded 1.2 mg/mL dispersions with 20 % monolayers and more than 90 % nanosheets less than 6 layers [34]. Transmission electron microscopy (TEM) of the dispersed sheets showed the systematic reduction of flake dimension with sonication time scaling as t^-1/2^. Concurrently, the graphene concentration (C_G_) steadily increased following the empirical relation of C_G_ α √t (Fig. 2(a)-(b)). In a similar experiment, 150 h bath sonication in DMF produced 1 mg/mL graphene suspensions consisting of predominantly few layer flakes, but unfortunately no information has been provided regarding the lateral size [35]. It is noteworthy that such a prolonged sonication is too much energy and time consuming for practical applications. Moreover, sonication induced scissions cause the lateral dimensions of the exfoliated graphene flakes to drop drastically. Generally, mild sonication of graphite for shorter time periods is regarded non-destructive, as the process leaves the graphene basal plane relatively unimpaired and if at all created, the defects would be principally located around the edges.

Recently, Coleman et al. reported high-shear mixing as a scalable alternative to sonication for the LPE of untreated graphite crystals. They demonstrated the scalability of the method to industrial manufacture level (Fig. 2(c)) [36]. Once the local shear rate exceeds 10^4^ s^−1^, exfoliation could produce large quantities (production rate as high as 0.4 g h^−1^) of defect-free, unoxidized graphene as indicated by the XPS and Raman spectroscopy.

### Purification: centrifugation

**Fig. 3:**
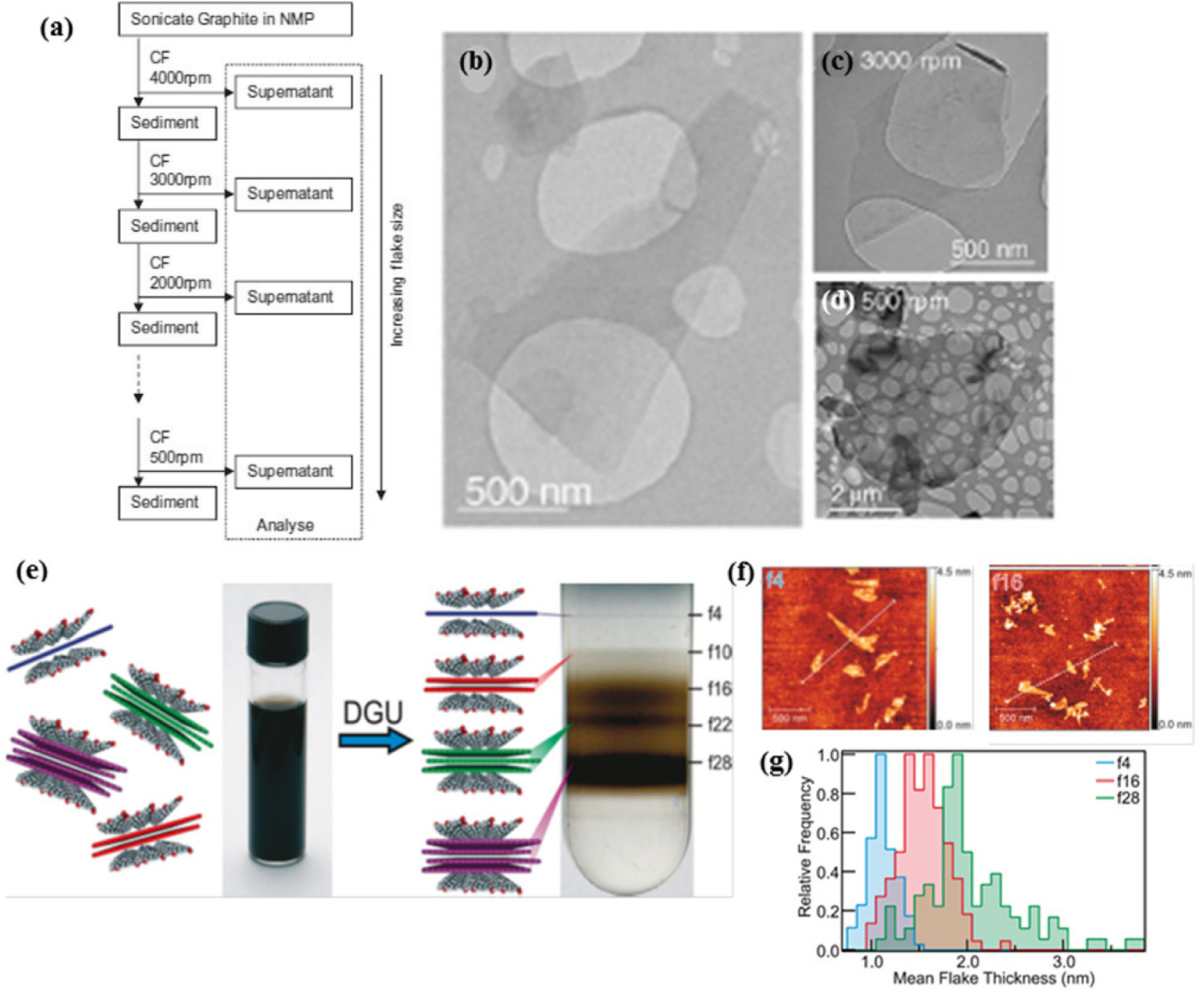
Centrifugal methods for purification of exfoliated graphene. **a** Schematic showing centrifugation based size selection procedure. (**b**-**d**) TEM images of exfoliated graphene sheets, (**b**) without any size selection after direct centrifugation at 500 rpm (**c**) with size selection centrifuged according to scheme (**a**) with a rate of 3000 rpm and (**d**) 500 rpm. **a**-**d** reproduced from ref. 37 with permission, © Elsevier). **e** Thickness sorting of graphene using density gradient ultracentrifugation (DGU). **f**-**g** Mean flake thickness histogram measured by AFM of sorted graphene taken from the locations marked in panel. **e**-**g** reproduced from ref. 38 with permission, © American Chemical Society)

The production of graphene dispersions by LPE inevitably causes a host of polydispersity and other material issues. It is well-known that the material properties of graphene significantly depend on the layer number. Therefore, once the graphite flakes are exfoliated, the next important step is the purification or separation of the exfoliated flakes from the un-exfoliated junk. Centrifugal processing is the most common technique used to separate monodisperse graphene suspensions, where sedimentation rate depends on the shape, size and buoyant density. When a polydisperse graphene suspension is subjected to high centrifugal force, graphene flakes with larger lateral areas sediment faster. As a result, when the centrifugation is completed, smallest flakes are found near the top of the centrifuge tube, whereas the larger flakes are located at the bottom. Direct exfoliation in solvents typically produces smaller graphene flakes within the size range of 1 μm, but majority of the applications require flake dimensions of at least few microns or above. Coleman et al. demonstrated controlled centrifugation as a versatile method for size-sorted fractionation of liquid phase exfoliated graphene dispersions with mean flake size varying from 1 to 3.5 μm [37]. As shown in Fig. 3(a), this method utilizes re-dispersion of sediment and low speed centrifugation cycles successively to produce different fractions with different mean flake size; 1 μm obtained at 4000 rpm and 3.5 μm at 500 rpm. Remarkably, the average number of graphene layers increased with decreasing centrifugation rate.

In many cases, it is difficult to decouple the effect of area and thickness polydispersity, which makes sedimentation-based centrifugal separation less useful. Under these conditions Green and Hersam succeeded in isolating monodisperse graphene dispersions according to their buoyant densities using density gradient ultracentrifugation (DGU) [38]. These thickness controlled graphene fractions were generated from sodium cholate encapsulated aqueous graphene dispersions, similar to that used for DGU separation of carbon nanotubes [39]. In DGU separation, the graphene dispersion is introduced to a density gradient designed with matching buoyant density distribution. These density gradients upon ultracentrifugation, moves the graphene sheets to their isopycnic points, where the buoyant density of graphene matches with that of the medium. Consequently visible bands appear in the centrifuge tube (Fig. 3(e)), signature of successful isopycnic separations. A monotonic increase in the thickness of the graphene with increasing buoyant density was observed from AFM measurements (Fig. 3(f-g)) along with selective enrichment of 1–4 layered graphene sheets. Samples with ∼ 85 % monolayer graphene have been produced using this process. In this approach, nonetheless, the density of the environment has to precisely match with that of the flake, which in turn would depend on both the thickness and lateral size of the flakes.

## Surfactant assisted liquid phase exfoliation

As mentioned in the introduction part, use of surfactants in the LPE of graphene is principally motivated to explore water as an exfoliating medium. By adding suitable surfactants, the high surface energy of water (72.8 mJ m^−2^) could be reduced and optimized to make a feasible interaction with highly hydrophobic graphitic surfaces. The first aqueous surfactant based exfoliation was reported by Lotya et al. using sodium dodecyl benzene sulfonate (SDBS) [40]. Following researches proved that surfactant assisted exfoliation can promote the stabilization of suspended graphene sheets against re-aggregation in organic solvents as well. A wide variety of ionic as well as non-ionic surfactants have been explored including both small molecules and polymers. Using non-covalent interactions, these surfactants interact with graphene surface by surface adsorption, micelle formation and/or π-π stacking. Ionic surfactants adsorbed onto graphene impart an effective charge, providing electrostatic repulsion to prevent re-aggregation of graphene sheets; meanwhile non-ionic surfactants provide the stabilization via steric interactions. We classified the entire range of surfactants into four main categories; (1) Aromatic and (2) Non-aromatic small molecules, (3) Ionic liquids and (4) Polymers, and discussed individually in the subsequent sections.

### Aromatic small molecule surfactants

#### Aromatic ionic surfactants

Aromatic small molecules can act as highly efficient surfactants because of their hydrophobic surfaces similar graphene and the strong π-π interactions between them can facilitate LPE process. SDBS, the first surfactant tested for graphite exfoliation, is also an aromatic ionic molecule with a polar sulfonate group and hydrophobic dodecyl chain attached to benzene ring [40]. A mixture of water, pristine graphite and SDBS were sonicated for 30 min, followed by centrifugation at 500 rpm for 90 min to produce 0.002 - 0.05 mg/mL suspensions. Small quantities (~3 %) of monolayer and large quantities (~43 %) of multi-layer (<5 layers) sheets were observed from the TEM and AFM analysis. Thin films prepared by the vacuum filtration of the as-obtained graphene suspensions showed a high sheet resistance (~970 kΩ/□) and conductivity (35 S/cm).

Hou et al. prepared aromatic anionic TCNQ (7,7,8,8-tetracyano-quinodimethane) coated graphene sheet suspensions in water as well as organic solvents [41]. The expanded graphite was mixed with TCNQ with a few drops of DMSO and the subsequent exfoliation was carried out in water in the presence of KOH to facilitate the reduction of TCNQ to harmful TCNQ anion. The exfoliated graphene sheets were principally 2–3 layer thick and lateral dimensions ranged from hundreds of nanometres to few micrometres. Notably, the Raman analysis of TCNQ adsorbed graphene showed an increased I_D_/I_G_ value compared with starting expanded graphite, which was attributed to the structural defects arising from the increased boundary edges of exfoliated sheets.

Charge transfer interactions between aromatic coronene salt and graphene were demonstrated by Rao et al. to exfoliate few layer graphene sheets prepared from thermally exfoliated graphite oxide (EG) and arc evaporated graphite in hydrogen atmosphere (HG) [42]. The starting materials EG/HG were mixed with the coronene surfactant and heated to 100 °C for 24 h, followed by a sonication at 70 °C for 2 h. Stable graphene suspensions with majority mono- and few-layer sheets were revealed by microscopic studies. Another aromatic amphiphilic molecule, Rose Bengal with a hydrophilic carboxylate group and hydrophobic aromatic framework was also found to be useful for exfoliation of microwave expanded graphite in 10 % DMA (N,N-dimethyl acetamide) aqueous solution [43]. More than 6 h bath sonication produced a mixture of mono- and few layer graphene dispersion with 12 wt.% yield and thin film prepared by vacuum filtration showed a high electrical conductivity of 12280 S/cm. Recently, Chen et al. showed direct exfoliation of HOPG (highly oriented pyrolytic graphite) using pyridinium tribromide (Py + Br3-) in 1:1 ethanol-water mixtures to give around 75 % monolayer sheets, which were stable over an year without any agglomeration [44]. In particular, the exfoliated flakes contained no significant defects as it was indicated by the absence of D-peak in the Raman spectra and exhibited notably high conductivity value of 5100 S/cm.

**Fig. 4:**
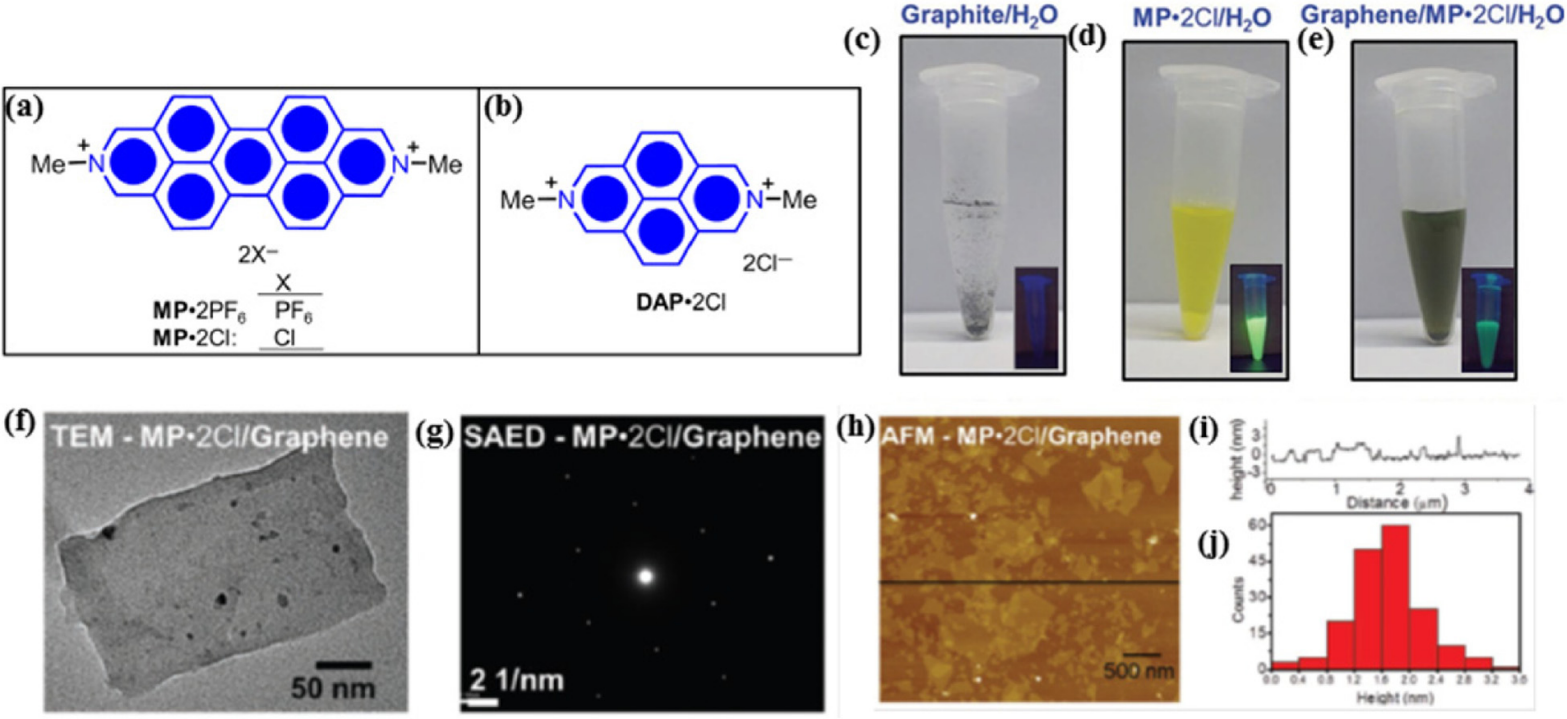
Diazaperopyrenium for effective graphene exfoliation. **a-b** Structural formulas of diazaperopyrenium dication (MP^2+^) **a** and diazapyrenium dication (DAP^2+^) (**b**). **c-e** Photographs of graphite/H_2_O(**c**), MP.2Cl/ H_2_O **d** and graphite/ MP.2Cl /H_2_O under ambient light and under UV light (insets). (**f**) TEM images of **MP** •2Cl/Graphene composite material. **g** SAED pattern of **MP** •2Cl/ Graphene. **h** AFM height image of **MP** •2Cl/Graphene. (**i**) Height profile of AFM image corresponding to the line shown in Fig. 4(**h**). **j** Probability of occurrence of graphene layers with various thickness measured by AFM height image. **a-j** reproduced from ref. 45 with permission, © Wiley-VCH)

Among many aromatic surfactants, the polycyclic aromatic hydrocarbons such as pyrene, perylene, anthracene etc. deserve special mention, as they can be considered as “nanographenes”. Majority of them have proved to be extremely efficient in reducing surface free energy of the graphene dispersion. These surfactants behave as molecular wedges that attach at graphitic surfaces via strong π-π stacking, which help cleavage into individual graphene sheets during ultrasonication or shearing. More the number of fused rings, better the exfoliation. For instance, in a very recent investigation Stoddart, Stupp and co-workers introduced a fused aromatic molecule, N,N’-dimethyl-2,9-diazaperopyrenium dication (MP^2+^) (Fig. 4(a)), which efficiently exfoliated graphite to graphene under mild sonication [45]. Depending on the counter ion, MP^2+^ could exfoliate graphite directly in water (MP.2Cl) as well as in organic solvents, such as DMF (MP.2PF_6_). Strong charge transfer interaction between MP^2+^ and graphene were demonstrated by fluorescence quenching studies as shown in Fig. 4(c-e). The graphene dispersion obtained after the removal of large graphitic particles was confirmed to consist of predominantly mono- and few layer sheets by combined Raman and microscopic studies. Even though no information has been provided regarding the yield of the process, the authors conducted a meaningful comparison of MP^2+^ with another dication DAP^2+^ (N,N’-dimethyl-2,7-diazapyrene) (Fig. 4(b)) which has 58 % less π-surface. Sonication of an aqueous mixture of DAP.2Cl and graphite even for more than 24 h could not induce any exfoliation. This control experiment clearly demonstrated the significance of extended π-conjugation to intercalate through the graphite layers and provide further stabilization via strong π-π interactions.

In recent years, commercially available pyrene derivatives with suitable polar functional groups have been used by large number of research groups, as stabilizers in graphene exfoliation. Commercial availability and high exfoliation efficiency compared to traditional surfactants are the principal motivations. Almost 90 % yield of monolayered graphene sheets was achieved by Dong et al. in 2009, by exfoliation of graphite powders with tetrasodium salt of 1,3,6,8-pyrenetetrasulfonic acid (Py-4SO_3_) [46]. In 2010, Zang et al. also reported aqueous phase exfoliation of graphite using 1-pyrenemethylamine hydrochloride (Py-NH_3_^+^) and 1,3,6,8-pyrenetetrasulfonic acid (Py-4SO_3_-) tetrasodium salt hydrate [47]. Fairly good quality few-layer graphene sheets with total oxygen content of 8.5 % and 16 % were obtained for Gr-Py-NH2 and Gr-Py-4SO3 hybrids, respectively, with nearly 50 % yield. In both the dispersions, positive and negative charges of the respective pyrene molecules adsorbed onto graphene surface provided static repulsive forces stabilizing the exfoliated sheets. More importantly, the pyrene derivatives acted as healing agents or electric “glue” during subsequent thermal annealing, where I_D_/I_G_ value of Raman spectroscopy changed from 0.64 to 0.46. Consequently, a high conductivity of 181200 S/m (778 Ω/□) and a light transmittance greater than 90 % were exhibited by the as-prepared graphene films, which is the highest conductivity value ever achieved for graphene films prepared by LPE (note that graphene films fabricated by the CVD method can reach 200 Ω/□ at 80 % transparency) [48]. Again in 2010, Kar and co-workers reported 1-pyrenecarboxylic acid (PCA) molecule assisted LPE of graphite powder, which had been commonly used to debundle single wall carbon nanotubes [49]. Graphite powder and PCA in methanol/water mixtures were sonicated for more than 24 h. Methanol was added to aid complete dissolution of amphiphilic PCA molecule. The non-covalent interaction of π-clouds produced graphene-PCA complex in 1 wt % yield, where the concentration of graphene in the final dispersions were around 0.01 mg/mL. The exfoliated graphene was a mixture of mono- and multilayer flakes. Nonetheless, the authors demonstrated highly sensitive and selective conductometric sensor application (whose resistance rapidly changes >10 000 % in saturated ethanol vapor), and ultracapacitors with extremely high specific capacitance (∼120 F/g), power density (∼105 kW/kg), and energy density (∼9.2 Wh/kg). In 2011, Rangappa and Honma et al. used 1-pyrene sulfonic acid sodium salt (Py-1SO3) in a novel one-pot in-situ supercritical fluid exfoliation of graphite in ethanol-water mixtures [50]. The presence of Py-1SO3 was shown to increase the mono- to bilayer graphene yield up to 60 % and also an increased Li-ion storage capacity was demonstrated compared to pure graphite materials.

**Fig. 5:**
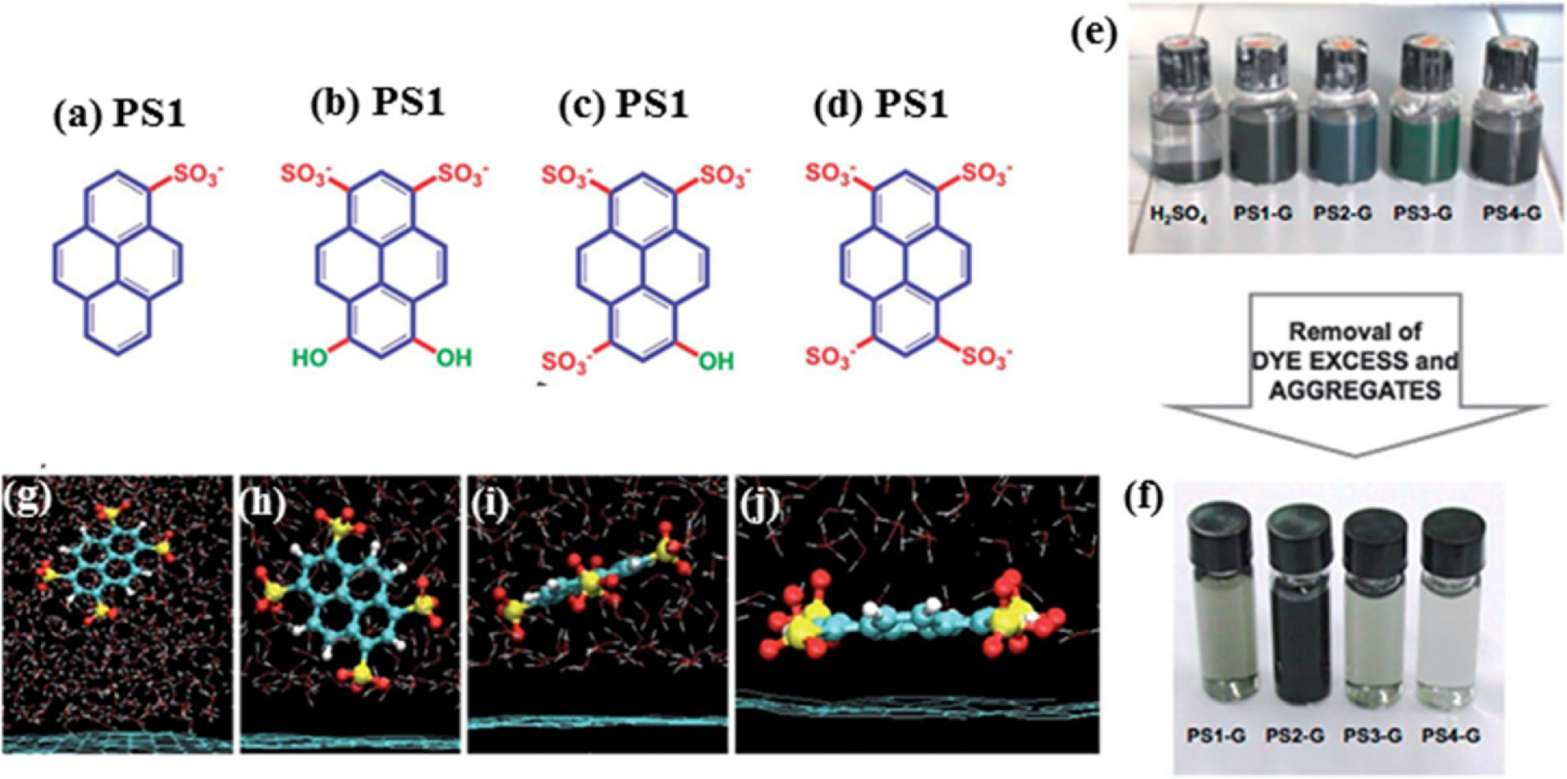
Pyrene dyes for high yield graphene exfoliation. **a-d** Chemical formulas of the 4 pyrene-sulfonate dye molecules studied for LPE in water. The protonated/deprotonated groups are indicated in green. **e** Photographs of 4 dye solutions after sonication with graphite and compared with that in concentrated sulphuric acid. (**f**) Images of the respective suspensions after removal of excess dye by washing and centrifugation indicating highest concentration obtained with PS2 surfactant. **g-j** Snapshots from molecular dynamic simulations of pyrene sulphonate molecules adsorbing on graphene in water. **a-j** reproduced from ref. 52 with permission, © Royal Society of Chemistry)

A bunch of different pyrene derivatives were compared by Green and co-workers as stabilizers for expanded graphite exfoliation, which included pyrene (Py), 1-aminopyrene (Py–NH_2_), 1-aminomethyl pyrene (Py–Me–NH_2_), 1-pyrenecarboxylic acid (Py-COOH), 1-pyrenebutyric acid (Py-BA), 1-pyrenebutanol (Py-BuOH), 1-pyrenesulfonic acid hydrate (Py-SAH), 1-pyrenesulfonic acid sodium salt (Py–1SO3) and 1,3,6,8-pyrenetetrasulfonic tetra acid tetra sodium salt (Py–4SO3) [51]. For all those pyrene derivatives the final graphene concentration increased initially with the addition of stabilizers and then decreased or remained constant (Fig. 5c); the highest yield obtained with Py-1SO3 which was around 0.8-1 mg/mL, whereas Py-4SO3 assisted exfoliation produced only 0.04 mg/mL few-layer graphene dispersion. Dispersions from pyrene stabilizers with sulfonyl functional groups also exhibited high temperature stability, hence promising for high temperature processing. Notably, the TEM images of Py-1SO3/Gr dispersion showed multi-layers and no information provided regarding the shelf-life of the dispersions.

Following the work by Green et al., in 2013 Palermo and co-workers provided a systematic comparative study on pyrenes exclusively with increasing sulfonyl groups by combining experimental and modelling investigations [52]. In addition to Py-1SO3 (or PS1) and Py-4SO3 (or PS4), they introduced two complex analogues 6,8-dihydroxy-1,3-pyrenedisulfonic acid disodium salt (PS2), 8-hydroxypyrene-1,3,6-trisulfonic acid trisodium salt (PS3) having electron accepting, sulfonic (−SO3-) groups and electron donating –OH groups (Fig. 5(a-d)). They found that PS2 derivative having the largest dipole and most asymmetric functionalization, produced dispersion with the highest graphene concentration. Molecular dynamic calculation revealed the involvement of a thin solvent layer between the dye and the graphene surface affecting the interaction. The amphiphilic molecule was found to change its orientation while approaching the surface to slide into this layer. Simulations indicated that the molecular dipole is thus not important per se, but because it facilitates the “sliding” of the molecule into the solvent layer, and therefore the lateral displacement of the water molecules collocated between the aromatic cores of the dye and the graphene substrate. Moreover, the stability and pH response of the suspensions showed no significant influence on the molecular charging and dipole. In another independent study, Casiraghi et al. made a comparison between Py-1SO3 and Py-4SO3 for exfoliation of graphite in water alone and no co-solvents [53]. Around 20 % monolayer was obtained with Py-1SO3 while graphene yield was very low in the case of Py-4SO3. The authors also pointed out the large discrepancy in results obtained by Dong et al. and Zhang et al. on LPE of graphite using Py-4SO3, thereby indicated the need for more detailed investigation on this topic. An overall comparison of exfoliation capability of the different aromatic ionic surfactants along with their structures is given in Table 1.

**Table 1:** Comparison of different aromatic ionic surfactants for producing colloidal dispersions of graphene

Surfactant/SA	Graphite source/Solvent	Sonication procedure/time	Yield/Gr-conc.	Flake lateral size	Thickness/quality	Shelf-life	Ref.
**SDBS**	Graphite powder Sigma/Water	Low power bath sonication (Branson 1510E-MT) 30 min	0.002-0.05 mg/mL	<1μm	Majority less than 5 layers. Exact value not given	35 % stable over 30 days	40
**TCNQ**	Expanded Graphite/Water	Sonication time 90 min.; type not mentioned.	0.015-0.02 mg/mL	100 nm –few μm	Majority 2-3 layers	NG	41
**Coronene carboxylate (CS)**	Thermally exfoliated graphite oxide (EG)(5L±1)/water	Heating Gr/CS mixtures at 100 ^o^C for 24 hrs. Sonication time 2 hrs at 70 ^o^C; type not mentioned.	HG-CS yield given 0.15mg/mL. EG-CS yield not mentioned	NG	0.5-1 nm thick 1-2layer flakes	NG	42
	Arc evaporated graphite in hydrogen atmosphere (HG)(3L±1)/water						
**Rose Bengal**	Expanded Graphite by microwave assisted heating/10 % Dimethyl-acetamide aqueous solution	Bath sonication 250W/6-10 hrs.	12 wt %	<400nm	>80 %,2-3layer flakes	NG	43
**Pyridinium tribromide (Py** ^**+**^ **Br** ^**3-**^ **)**	HOPG	Bath type sonicator(Branson® 3510R-DTH)/45 min	0.04 mg/mL	sub μm to several μms	Average thickness 174±105 nm. 75 % single layer	Over 1year	44
**Diazaperopyrenium dication MP** ^**2+**^	Graphite powder (Alfa Aesar)/ DMF, Water	Sonication time 30 min; type not mentioned	NG	NG	2-4 layers majority	More than 3 weeks	45
**Diazapyrenium dication DAP** ^**2+**^	Graphite powder (Alfa Aesar)/ DMF, Water	Time 24 hrs	No exfoliation at all.				45
**Pyrene-carboxylic acid (Py-COOH)**	Graphite powder (Sigma)/ Methanol-water (1:4)	Bath sonicator (Branson 5510) 45 min sonication in MeOH, 24 hrs sonication in MeOH/H_2_O.	0.01 mg/mL	100 nm to few μm	Less than 10 nm thick few layers.	>10 days	49
**Pyrene-NH**_**2**_	Synthetic graphite (<20 μm) (Sigma)/Water	Bath Sonicator (Sonics VX-130, 130W, 45 % power)ice bath, 2hrs	50 %	μm range,	Average thickness 0.9±3 nm	2 days	47
**Pyrene-4SO**_**3**_	Synthetic graphite (<20 μm) (Sigma)/water	Less than 2 hrs	NG	NG	Average thickness 1.3 -2.6 nm	NG	47
**Pyrene-1SO**_**3**_	Graphite powder /Ehtanol-water (5:1)	Bath sonication (US-4R, 40KHz, 10W)/30 min, followed by heating at 450 ^o^C for 2h with SCF shaking.	60 %	1-1.5 μm	0.6-2 nm 60 % 1-2layers.	NG	50
**Pyrene-1SO**_**3**_	Expanded Graphite (Asbury Carbons CAS 7782-425 ,GRADE-3805)/DI-water	Tip sonication(Misonix-XL2000, 7W)/1hr.	0.8-1 mg/mL	2-2.5 μm	2-4layers	NG	51
**Pyrene-4SO**_**3**_	Graphite powder (NGS-Germany)/D_2_O solvent	70 W Probe sonicator(pulse mode in ice bath)/2hrs	NG	NG	1.29-1.65 nm 90 % single layer	NG	46

#### Aromatic non-ionic surfactants

**Fig. 6:**
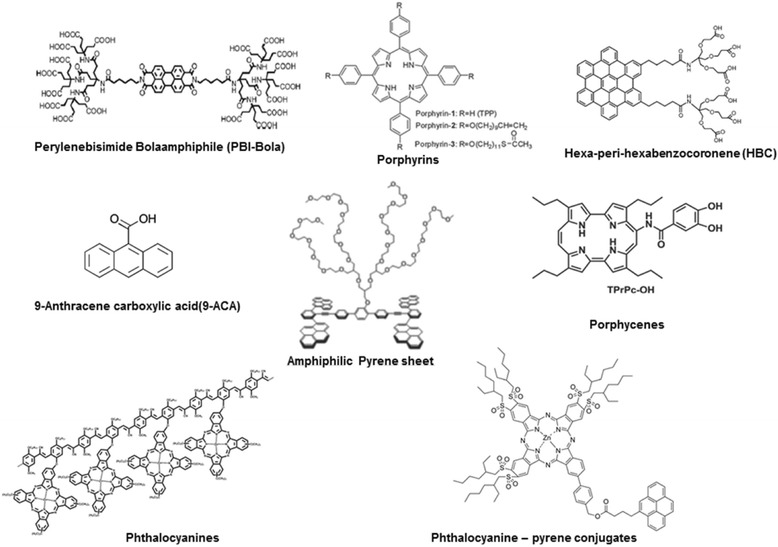
Chemical structures of aromatic non-ionic surfactants described in the section 3.1.2

In addition to the ionic variations, several non-ionic aromatic stabilizers (Fig. 6) have also been explored. For instance, a water soluble perylenebisimide bolaamphiphile (PBI-Bola) was synthesized by Hirsch and co-workers as a graphite exfoliant [54]. More than 6 h ultrasonication of graphite powder in the aqueous phosphate buffer (P^H^ = 7.0) solution of this perylene detergent generated a polydisperse mixture of monolayer and few layer graphene sheets. Jung et al. dispersed graphite in the solutions of different porphyrins like 5,10,15,20-tetraphenyl-21H,23Hporphine(TPP, porphyrin-1) and its derivatives containing functionalized alkyl groups at the para-positions of benzene rings (porphyrin-2, −3,) in NMP containing organic ammonium ions, such as tetrabutyl ammonium hydroxide (TBA) [55]. Graphite dispersion generated from porphyrin-3/graphite/TBA/NMP provided high quality graphene sheets as demonstrated from TEM and Raman analysis. Nearly 5 times higher graphene concentration (0.05 mg/mL) was obtained compared to the exfoliation in NMP alone, but no report on the overall yield of the process. Guldi and co-workers have pioneered the use of porphycenes, phthalocyanines, porphyrins,phthalocyanine-pyrene conjugates etc. in LPE of graphite and realized monolayer/bilayer nanographene charge transfer hybrids [56–60]. Very recently, Muellen et al. reported the synthesis of an amphiphilic hexa-peri-hexabenzocoronene molecule, which could assist the LPE in polar solvents such as methanol [61]. Graphene dispersion with the concentration as high as 1.1 mg/mL containing 2–6 layer nanosheets was successfully achieved. 9-Anthracene carboxylic acid (ACA), another amphiphilic aromatic hydrocarbon was successfully used by Lee et al. for LPE with the aid of non-covalent functionalization [62]. Ultrasonication for more than 24 h was carried out in ethanol/water mixture to achieve stable aqueous ACA-Gr dispersions in 2.3 % yield. Ultracapacitor based on these dispersions showed good specific capacitance of 148 F/g. An amphiphilic aromatic molecular sheet based on four pyrene units and a laterally grafted hydrophilic oligoxyethylene dendron was designed by Lee and co-workers for selective dispersion of 2-D graphene sheets in MeOH/water solution [63]. This method reported a very high final graphene concentration of 1.5 mg/mL, with pre-dominant mono- and bilayer exfoliation argued on the basis of microscopic measurements. Unfortunately, very few reports provided systematic analysis on statistical distribution of thickness and lateral dimension of the exfoliated graphene sheets to figure out the exact scalability of each method.

#### Non-aromatic surfactants

**Fig. 7:**
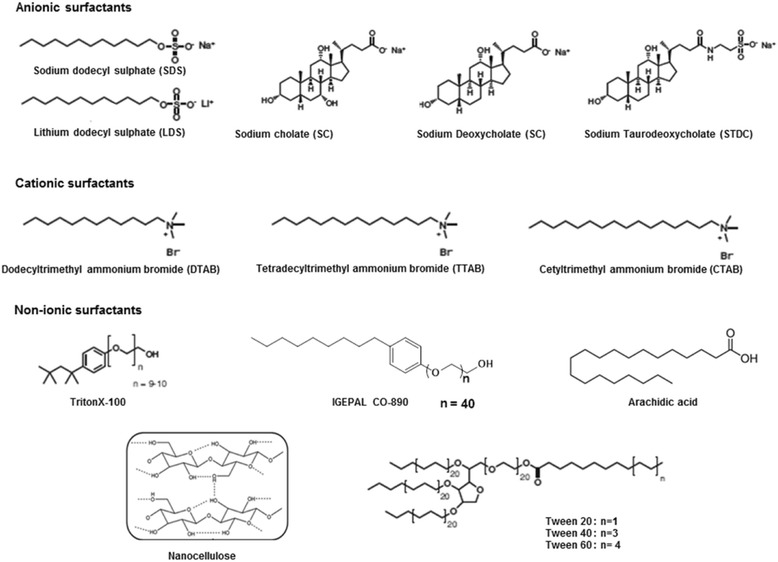
Chemical structures of non-aromatic surfactants described in the section 3.2

The chemical structures of several non-aromatic surfactants are given in Fig. 7. Valiyaveetil et al. directly exfoliated HOPG using a cationic surfactant CTAB (cetryltrimethyl ammonium bromide) in acetic acid to produce graphene nanoflakes of average 1.18 nm thickness, which also showed good dispersability in organic solvents like DMF [64]. Field emission properties of the graphene flakes demonstrated the turn on voltage of 7.5 V/μm and emission current density 0.15 mA/cm^2^. Sodium cholate (SC), which is a well-known efficient surfactant for carbon nanotubes, was employed by Coleman group for LPE of graphene in a procedure similar to that using SDBS [65]. However, extensively long 430 h of ultrasonication could produce only ~0.3 mg/mL concentrated graphene dispersions in water/SC mixture. Free standing graphene films with average conductivity of 17500 S/m were obtained after 2 h thermal annealing at 500 °C in an Ar/H_2_ atmosphere. Green and Hersam also attempted sodium cholate/water exfoliation using high intensity ultrasonic horn and ended up with a mixture of polydisperse graphene solution of 0.09 mg/mL concentration. Nevertheless, they successfully separated the dispersion according to layer thickness using density gradient ultracentrifugation. In a rigorous study, Smith et al. investigated twelve non-aromatic different types of ionic and non-ionic surfactants for aqueous exfoliation [66]. Final graphene concentration varied significantly; 0.026 mg/mL for sodium cholate and 0.011 mg/mL for sodium dodecyl sulfate. Meanwhile, there was a very little variation in the dispersed flake size and thickness.

Samori et al. recently demonstrated a long chain aliphatic fatty acid, arachidic acid that exhibits a high selectivity to graphene surface attachment, so as to act as dispersion-stabilizing compound for LPE [67]. High concentration conductive graphene ink was prepared following this supramolecular strategy and, thus, opened up new avenues for cost effective technological applications. Relevant reports motivated from this work are rapidly growing with many suggestions for the potential surfactants for low cost exfoliation, some of which include Gum Arabic [68], organosilanes [69], cellulose nanocrystals [70] etc.

#### Ionic liquids

**Fig. 8:**
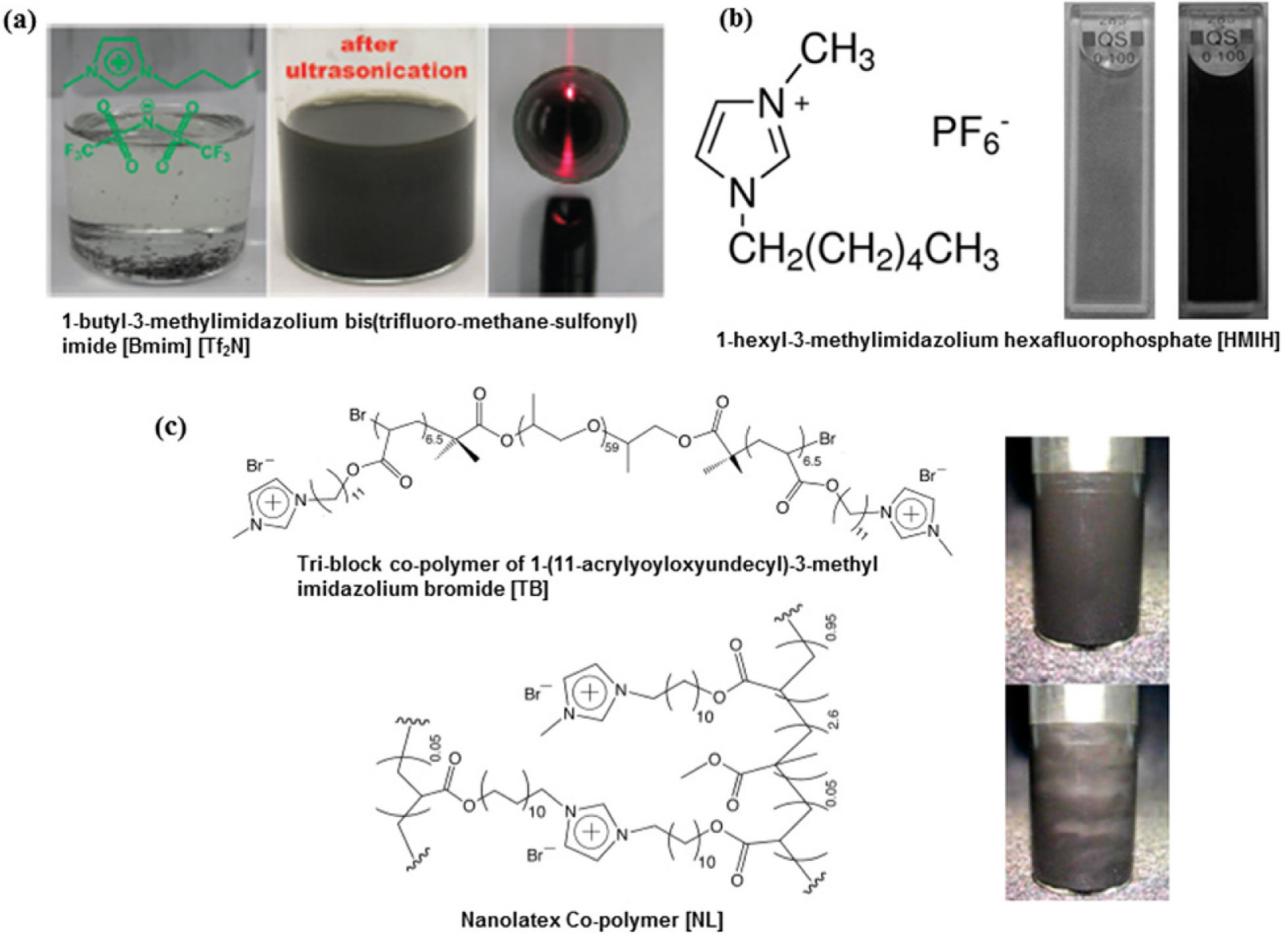
Ionic liquids assisted LPE of graphene. **a** Images of the dispersion of graphite in [Bmim] [Tf2N] before (left) and after (middle) ultrasonication and the Tyndall effect of a diluted graphene suspension using a laser pointer (right). (reproduced from ref. 73 with permission, © Royal Society of Chemistry). **b** HMIH structure and images of dispersions obtained after 0.5 h (left) and 24 h (right) of sonication time for samples with 1 wt % of initial graphite using HMIH. (reproduced from ref. 74 with permission, © Royal Society of Chemistry). **c** Triblock (TB), Nanolatex (NL) co-polymer structures and NL stabilized graphene rheo-optical dispersion (1.1 wt %) exhibiting isotropic to nematic transition upon application of shear field. (reproduced from ref. 75 with permission, © American Chemical Society)

Ionic liquids (IL) are semi-organic molten salts, composed of ions, which exhibit highly viscous liquid behaviour [71]. ILs generally have high capability to dissolve a wide range of solutes and they are also recyclable. Their miscibility and high viscosity can be tuned via the chemical modification of counter ions [72]. Moreover, ILs have high electrical conductivity and often exhibit surface energies close to graphene. Another interesting property that makes ILs promising for the exfoliation of graphene is their ionicity, a highly favourable feature that can stabilize the exfoliated graphene sheets via Coloumbic repulsive forces. In 2010, Wang et al. reported the first IL assisted exfoliation of natural graphite flakes using 1-butyl-3-methyl-imidazolium bis(trifluoromethane sulfonyl) imide ([Bmim] [Tf_2_N]) (Fig. 8(a)) [73]. Tip ultrasonication of ([Bmim] [Tf_2_N])/graphite mixture for 1 h afforded a high concentration (0.95 mg/mL) stable suspension of un-oxidised few-layer (≤5 layers) graphene sheets with micrometre-long lateral dimensions. Later in 2011, Nuvoli et al. reported an unprecedented graphene concentration as high as 5.33 mg/mL by sonicating a commercially available IL 1-hexyl-3-methyl imidazolium hexafluorophosphate (HMIM) (Fig. 8(b)) with graphite up to 24 hrs [74]. Nonetheless, this study lacks a detailed quantitative analysis; noteworthy the suspensions contained mixture of mono-, bi- and few-layer graphene sheets with average thickness of 2 nm and some of the flakes were around 4 μm.

Very recently, Texter and co-workers developed two excellent water stabilizers for graphene viz., triblock (TB) copolymer and copolymer nanolatex (NL) (Fig. 8(c)) based on a reactive IL acrylate surfactant 1-(11-acrylyoyloxyundecyl)-3-methyl imidazolium bromide (ILBr) [75]. Surprisingly, this method claim essentially complete exfoliation without the need of centrifugation to eliminate any undispersed contents and could produce graphene aggregates in water at concentrations upto 5 w %. They demonstrated that these graphene dispersions were rheo-optical fluids and simple Couette shear fields could align submicron-micron sheets over macroscopic areas indicating its bright future for surface coating applications. Moreover, the work also illustrated the transfer of graphene sheets in water to non-aqueous media with the aid of stimuli responsiveness to various anions. Despite all these advantages, the procedure adopted high power and remarkably long sonication time upto 113 h, which has led to dramatically reduced flake dimensions, as confirmed by SEM. Nevertheless, given the very high graphene concentrations obtained by this protocol, ILs deserve more detailed investigation for LPE, even though the yield of monolayers seem to be unclear yet.

#### Polymeric surfactants

Researches on polymer stabilized LPE has expanded realms such that it is impossible to consider all of them in this confined discussion. It is noteworthy that the resulting graphene/polymer composites commonly exhibit novel synergistic properties, which are unknown in the individual components. In this section, we will provide only a brief discussion on some of the most highlighted investigations.

**Fig. 9:**
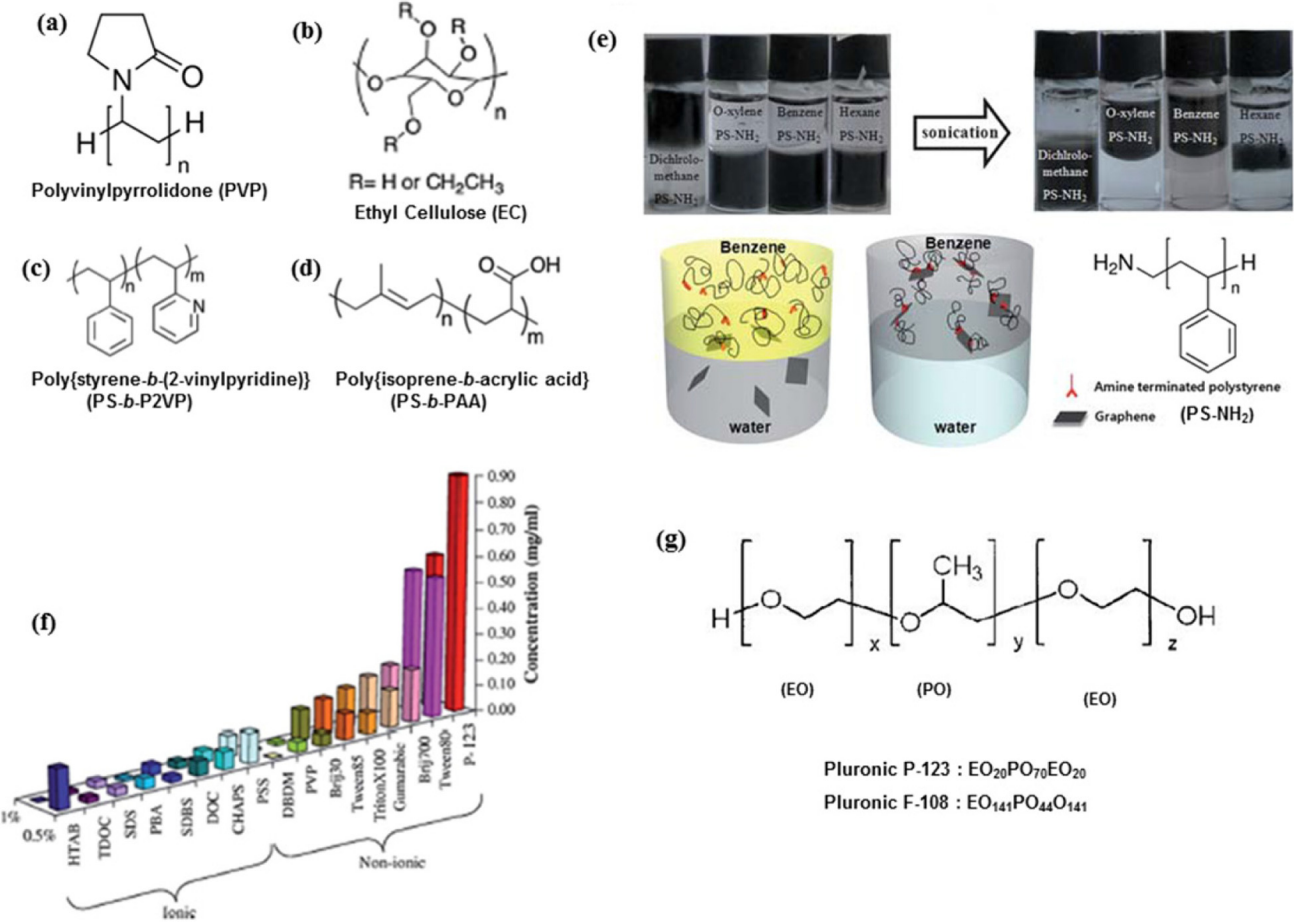
Polymeric surfactants in LPE. **a-d**,**g** Chemical structures of polymers described in the section 3.4. **e** Phase transfer of graphene from aqueous phase to organic phase via non-covalent PS-NH_2_ functionalization. (reproduced from ref. 76 with permission, © Royal Society of Chemistry). **f** Histogram comparing graphene concentration obtained by different non-ionic and polymeric surfactants. (reproduced from ref. 89 with permission, © Elsevier)

In terms of exfoliation procedure, polymer-mediated exfoliation is similar to other surfactant assisted LPE, but the key difference is in the exfoliated sheet stabilization mechanism. Colloidal stability of the most polymer-exfoliated graphene suspensions are provided by steric factors in combination with non-covalent interactions. From the early day research, covalent and non-covalent functionalizations of exfoliated graphene sheets with polymer chain have been utilized for colloidal stability in solvent media. As an example, our research group reported a novel non-covalent functionalization of graphene using end-functional polymers to achieve stable dispersions in several organic media [76]. Aqueous dispersions of reduced graphene oxide were non-covalently functionalized with amine terminated polystyrene (PS-NH_2_) to facilitate the phase transfer of graphene sheets from water phase to organic phase via simple sonication (Fig. 9(e)). It was found that various other end-functional polymers including PS, PMMA-OH, PS-COOH failed to provide efficient organo-dispersibility of reduced graphene in benzene compared to PS-NH_2_. This control experiment along with FTIR and Raman spectroscopy investigations verified the significance of non-covalent interaction between protonated amine terminal group of PS-NH_2_ and free carboxylate groups at graphene surface, driving the high dispersibility in various organic solvents. Direct growth of polymer brushes from exfoliated graphene surface was also used to solubilize graphene in desired solvents. Covalently functionalized graphene oxide was used as a macro-initiator, wherein different types of polymer brushes including polystyrene, poly methylmethacrylate or poly butylacrylate were attached onto graphene surface via atom transfer radical polymerization (ATRP) [77]. This kind of modification proved that polymer functionalization is greatly advantageous for the colloidal stability of exfoliated graphene sheets in many different solvent systems.

There are many studies on the exfoliated graphene composites based on a wide range of polymers, for instance, polystyrene (PS) [78], poly(styrene-co-butadiene-co-styrene) [79], poly methyl methacrylate (PMMA) [80], polyetherimide (PEId) [81], polylactide (PLA) [82], polypropylene [83], cellulose acetate [84], hyperbranched polyethylene (HBPE) [85] and so on. Since graphene is highly hydrophobic in nature, organic solvents are much more compatible for LPE, but water appears to be a more appealing choice when it comes to a cheaper and non-toxic green solvent for scalable processing. Such a hydrophobic to hydrophilic switching of graphite surface was achieved by Bourlinos et al. without any oxidation or damage to the sp^2^ carbon framework of graphene [86]. They chose polyvinylpyrrolidone (PVP) (Fig. 9(a)), a non-ionic and biocompatible polymer surfactant for the straightforward LPE of graphene in aqueous phase under mild sonication for about 9 hrs. Specifically, PVP was chosen owing to its high solubility in water and great affinity to graphite surface; another reason was that PVP contains N-substituted pyrrolidone ring structure similar to NMP solvent, an efficient graphene exfoliant. Stable aqueous dispersions of the hydrophilic polymer coated graphene monolayers were obtained in 10 - 20 % yield, as confirmed by the AFM, TEM and Raman spectroscopy. The colloidal stability of the exfoliated graphene layers in water was suggested to be conferred by steric or/and depletion stabilization by the non-ionic yet largely hydrophilic polymer. Tagmatarchis and c-workers applied another trick to switch the solubilty of graphene from organic to water phase [87]. They exfoliated graphene sheets in organic solvents such as NMP and *o*-DCB. Subsequent treatment of the exfoliated sheets with an acidic solution of poly[styrene-b-(2-vinylpyridine)] (PS-*b*-P2VP) (Fig. 9(c)) or poly(isoprene-*b*-acrylic acid) (Fig. 9(d)) (PI-b-PAA) block copolymers switched the dispersability into aqueous solutions.

Efficient exfoliation of graphene in a non-traditional solvent, ethanol was achieved by Hersam and Liang, by the addition of ethyl cellulose (Fig. 9(b)) as a stabilizing polymer [88]. The post sedimentation graphene concentration in ethanol was found to increase from 1.6 to 122.2 μg/mL after 3 h sonication in the presence of ethyl cellulose. In an attempt to increase the dispersibility even further, the authors also developed an iterative solvent exchange using terpineol, ultimately yielding stable graphene inks to a level exceeding 1 mg/mL. Highly aligned graphene-polymer composites solution-cast from these inks demonstrated outstanding processability, and transparent conductive graphene thin films were also successfully prepared. In a rigorous study, Guardia et al. compared a wide range of ionic and non-ionic surfactants including polymers [89]. Their findings signalled that non-ionic surfactants especially polymers outperformed the ionic counterparts for the high yield production of defect-free graphene. (Fig. 9(f)) The highest concentration of ~1 mg/mL was achieved by sonicating graphite with a triblock copolymer, Pluronic®P-123 (0.5 % w/v) for just 2 hrs and extending sonication time to 5 hrs afforded 1.5 mg/mL dispersions (Fig. 9(g)). AFM images of the graphene samples on SiO_2_/Si showed an average flake thickness of 1.0 - 3.0 nm. Defect-free basal planes of the vacuum filtrated graphene films were revealed by STM imaging and these films exhibited high conductivities (1160 S/m) as well. Notley, in a similar study, compared pluronic non-ionic surfactants, F108 (molecular weight ~ 14.6 kDa) and F127 (molecular weight ~ 12.5 kDa) with some ionic surfactants such as CTAB (hexadecyltrimethylammonium bromide), TTAB (tetradecyltrimethylammonium bromide), DTAB (dodecyltrimethylammonium bromide) and SDS (sodium dodecylsulfate,) [90]. Interestingly, there was a key difference in the exfoliation procedure adopted by Notley compared to other procedures. A continuous surfactant addition method was employed during the sonication rather than adding all the surfactant at once before sonication. The idea was to continually maintain optimum surface tension of the surfactant/graphene solution by replacing the depleted surfactant that goes adsorbed on to graphene surface, throughout the sonication period. Graphene suspensions with very high concentrations of up to 1.5 % w/w (15 mg/mL) were achieved by the continuous addition of a highly concentrated aqueous solution of Pluronic F108 to graphite/water mixtures.

**Fig. 10:**
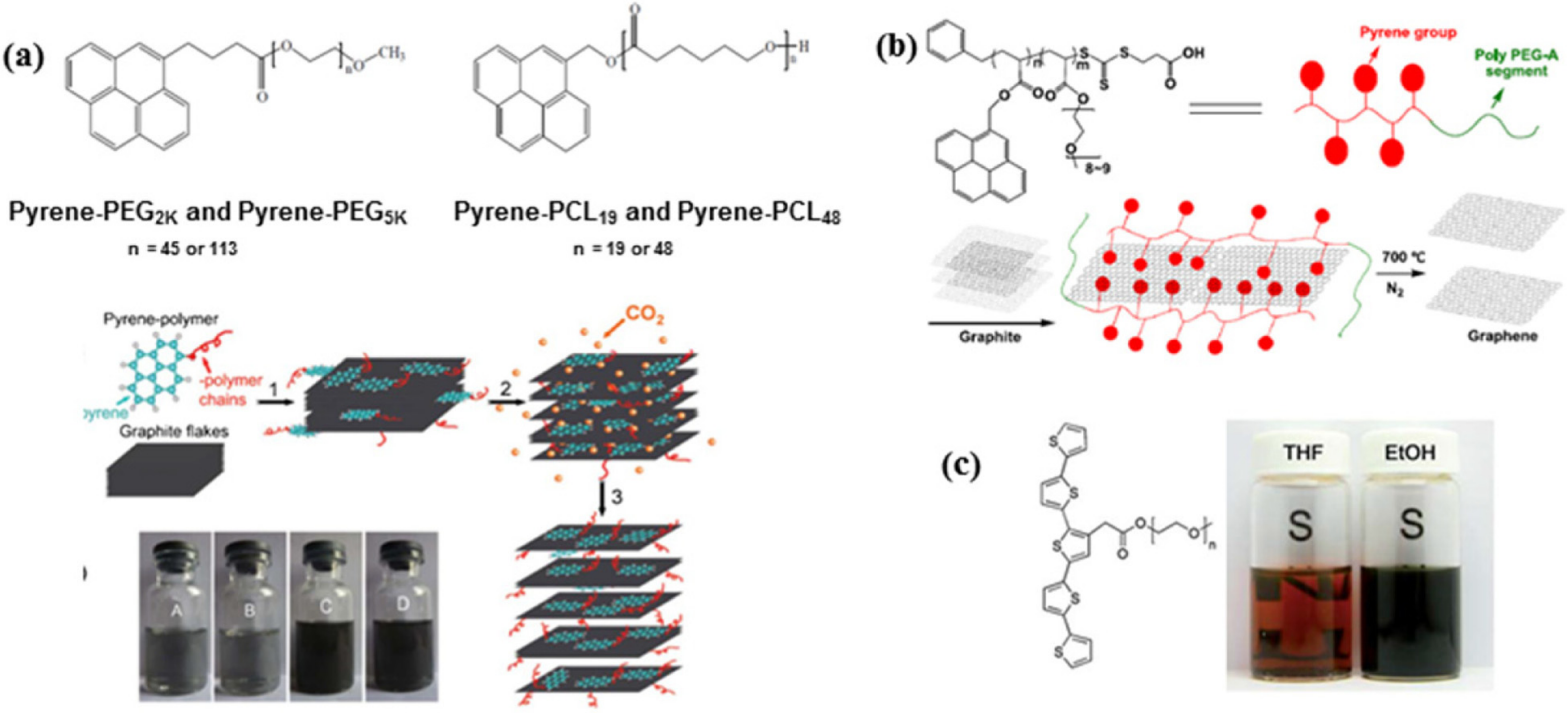
Pyrene and thiophene polymers in LPE. **a** Chemical structures of pyrene -PEG and PCL polymers and schematic illustration of the preparation process of pyrene polymers functionalized grapheme sheets based on SC CO_2_’s assistance (from step 1 to step 3) with images of pyrene polymer-functionalized graphene dispersions. (reproduced from ref. 91 with permission, © Royal Society of Chemistry). **b** Structure of pendant multi-pyrene polymer synthesized by RAFT along with schematic showing direct exfoliation of graphene. (reproduced from ref. 92 with permission, © Elsevier). **c** Chemical structure of 5TN-PEG and comparison of graphite-5TN-PEG dispersion in THF and ethanol. (reproduced from ref. 94 with permission, © Royal Society of Chemistry)

As mentioned above, Pyrene derivatives have been widely investigated as small molecule conjugate stabilizers. Concurrently, a few reports on pyrene-based polymers are also exploited as efficient graphene exfoliant. One typical report is from Zheng et al., wherein supercritical (SC) CO_2_ has been described as an effective medium for the direct LPE of pyrene-polymers stabilized graphene sheets with a good aqueous and organic solvent dispersability [91]. Specially synthesized pyrene-terminated polymers, pyrene-polyethylene glycol (Py-PEG_2K_ and Py-PEG_5K_) and pyrene-polycaprolactone (Py-PCL_19_ and Py-PCL_48_) (Fig. 10(a)), were used to exfoliate and stabilize graphene. The exfoliation procedure was carried out by sonicating Py-polymer/graphite mixture in DMSO for 3 h, followed by the exposure to SC/CO_2_ binary medium for 6 h and further additional sonication for 2 h. With the assistance of SC/CO_2_, pyrene-polymers were proposed to act not only as molecular wedges to cleave graphite to obtain graphene, but also as a modifier to functionalize exfoliated graphene to form stable dispersion in water and organic solvents, depending on the dangling polymer chains. The yields of exfoliated graphene sheets reached as high as 10.2 % in water and 51.8 % in DMSO, with a mixture of mono-, bi-or tri- and multi-layered sheets. In another investigation, Yang et al. prepared one-step graphene/polymer nanocomposites by successful straightforward exfoliation of micro-sized graphite in a pyrene-functionalized amphiphilic block copolymer matrix viz., poly(pyrenemethyl acrylate)-*b*-poly[(polyethylene glycol) acrylate] (polyPA-*b*-polyPEG-A) (Fig. 10(b)) in either aqueous or organic media [92]. PolyPA-*b*-polyPEG-A was prepared by (RAFT) polymerization and graphite powder mixed with different amounts of aqueous copolymer solutions were sonicated for 6 h at 30 °C to provide upto 78 % graphene yield when the copolymer to graphite ratio reached 40. This amphiphilic polymer design with multi-pyrene groups was proposed to bind at the graphite surface simultaneously via π-π stacking, working like “sucking discs” to drag the bound surface graphene sheet off the graphite precursor with the assistance of sonication. The as-prepared graphene/polymer composite films also exhibited increased tensile strength and tunable conductivity. Direct exfoliation of graphite flakes in the presence of pyrene-labeled single stranded DNAs yielded highly aqueous dispersible mono- and bi-layer graphene sheets with 100 nm to 4 μm flake size [93]. Subsequently, graphene-Au nanoparticle nanocomposites were produced by hybridizing the DNA immobilized at graphene surface with Au nanoparticle labeled complementary DNAs.

Highly conductive and transparent graphene films were fabricated by Jo et al. from direct exfoliation of graphite using a non-ionic semiconducting polymer quinquethiophene-terminated PEG (5TN-PEG) (Fig. 10(g)) as a surfactant in ethanol solution [94]. Washing off the excess surfactants by THF from the vacuum filtered films, followed by chemical treatment with nitric acid and thionyl chloride, resulted in a very low sheet resistance of 0.3 kΩ/□ with 74 % transmittance at 550 nm. This is one of the lowest values of sheet resistance among graphene films prepared by top-down fabrication.

## Conclusion and outlook

It is now well-recognized that one critical bottleneck standing in front of commercial utilization of graphene is the lack of a reliable mass production method for high quality graphene. In this context, LPE has long been considered as one of the most promising and versatile approach. In this review article, we highlighted the recent research progress in the production of high quality graphene by LPE, with a particular emphasis on the versatile role of different categories of surfactants.

LPE of graphene was initially developed with specific surface energy matching solvents (without surfactant). Relevant crucial processing factors such as solvents, external forces like ultrasonication/shear and purification methods by centrifugation have been discussed in detail in association with their influences on the exfoliation results. Significantly, most of the solvents used in the initial studies had revealed significant drawbacks, such high toxicity, high boiling point etc. that prompted the re-direction of research into environment benign less toxic solvents like water. Unfortunately, the surface energy of pure water is too high for graphene exfoliation such that a variety of surfactants have been introduced thus far. We categorized the large spectrum of surfactants in accordance to their structural functionalities into aromatic, non-aromatic, ionic liquids and polymers. Innumerable surfactants have been studied in this regard and many of them appeared highly promising LPE results.

For the further progress of surfactant assisted LPE, several shortcomings must be overcome: (1) The overall yield of LPE is still low; (2) Good exfoliating solvents are expensive and harmful; (3) Sonication/Shearing commonly lead to the drastic reduction in the size of exfoliated graphene sheets; (4) Residual surfactants are difficult to remove; (5) Typical surfactants are electrically insulators, which may significantly deteriorate the electrical connectivity among graphene layers; (6) All LPE methods produce graphene sheet with a high polydispersity in terms of lateral size as well as thickness. The future of real-life graphene applications strongly depends on how materials scientists address these formidable challenges and establish ideal large-scale LPE process for high quality graphene sheets. It is also highly required to attain more fundamental and systematic understanding of the exfoliation mechanism for innovative design of LPE schemes.
